# Improving drug-induced liver injury prediction using graph neural networks with augmented graph features from molecular optimisation

**DOI:** 10.1186/s13321-025-01068-3

**Published:** 2025-08-18

**Authors:** Taeyeub Lee, Joram M. Posma

**Affiliations:** Section of Bioinformatics, Division of Systems Medicine, Department of Metabolism, Digestion and Reproduction, Imperial College London, London, W12 0NN UK

**Keywords:** Drug induced liver injury, Feature engineering, Graph neural networks, Molecular optimisation

## Abstract

**Purpose:**

Drug-induced liver injury (DILI) is a significant concern in drug development, often leading to the discontinuation of clinical trials and the withdrawal of drugs from the market. This study explores the application of graph neural networks (GNNs) for DILI prediction, using molecular graph representations as the primary input.

**Methods:**

We evaluated several GNN architectures, including Graph Convolutional Networks (GCNs), Graph Attention Networks (GATs), Graph Sample and Aggregation (GraphSAGE), and Graph Isomorphism Networks (GINs), using the latest FDA DILI dataset and other molecular property prediction datasets. We introduce a novel approach that creates a custom graph dataset, driven by molecular optimisation, that incorporates detailed and realistic chemical features such as bond lengths and partial charges as input into the GNN models. We have named our model approach DILIGeNN.

**Results:**

DILIGeNN achieved an AUC of 0.897 on the DILI dataset, surpassing the current state-of-the-art model in the DILI prediction task. Furthermore, DILIGeNN outperformed the state-of-the-art in other graph-based molecular prediction tasks, achieving an AUC of 0.918 on the Clintox dataset, 0.993 on the BBBP dataset, and 0.953 on the BACE dataset, indicating strong generalisation and performance across different datasets.

**Conclusion:**

DILIGeNN, utilising a single graph representation as input, outperforms the state-of-the-art methods in DILI prediction that incorporate both molecular fingerprint and graph-structured data. These findings highlight the effectiveness of our molecular graph generation and the GNN training approach as a powerful tool for early-stage drug development and drug repurposing pipeline.

Scientific Contribution: DILIGeNN is a GNN framework that extracts graph features from 3D optimised molecular structures as is done in target-based drug discovery and molecular docking simulation. Our method is the first to encode spatial and electrostatic information into a single graph representation, as opposed to other work that require multiple graphs or additional chemical descriptors for feature representation. Our approach, using warm starts following repeated early stopping during training, outperforms the current state-of-the-art methods in liver toxicity (DILI), permeability (BBBP) and activity (BACE) prediction tasks.

**Graphic Abstract:**

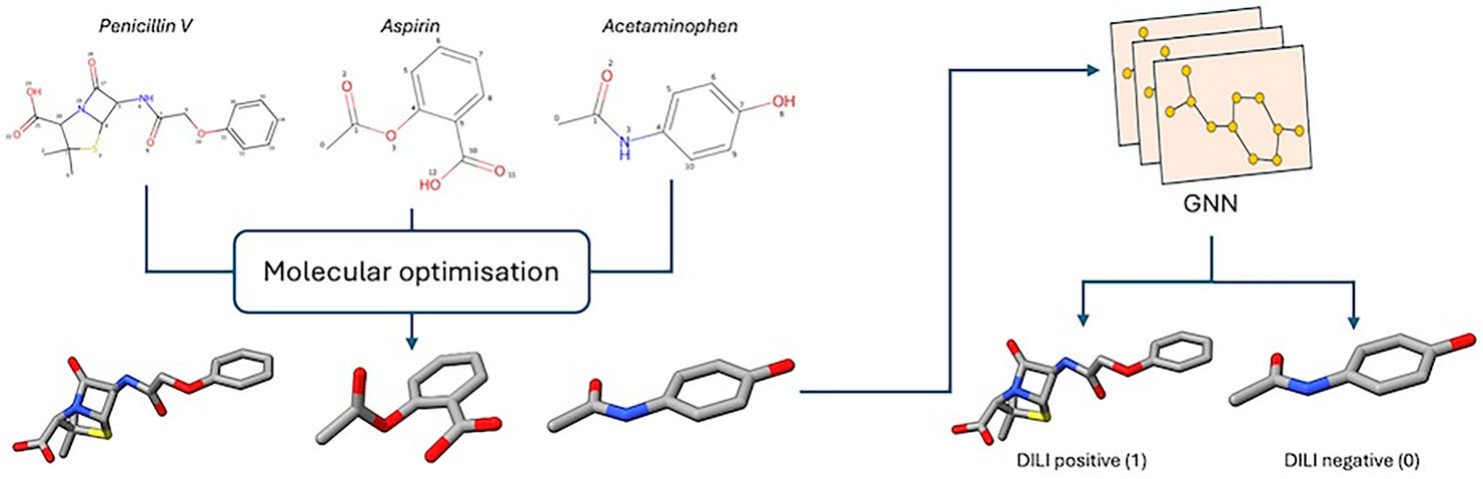

## Introduction

Drug-induced liver injury (DILI) refers to the unexpected liver damage caused by clinical drugs, affecting hepatocytes and other liver cells. It is a major concern in drug development, often causing clinical trials to be halted and marketed drugs to be withdrawn due to severe impacts on patient safety [1, 2]. DILI accounts for approximately 50% of drug-induced acute liver failure cases and is linked to more than 1,000 FDA approved drugs [3, 4].

Drug-induced liver toxicity is broadly classified into two types; idiosyncratic and intrinsic. While intrinsic DILI is typically dose-dependent and can be predicted based on the drug’s pharmacological properties, idiosyncratic DILI occurs unpredictably and does not correlate with the dosage [5]. The idiosyncratic DILI often have a prolonged latency period, with liver toxicity appearing weeks to months after the initiation of therapy [6].

Its detection at an early pre-clinical stage is challenging due to the low concordance between animal models and human outcomes, with concordance rates of only 63% in non-rodent and 43% in rodent models [7]. In addition, despite the advances in *in vitro* assays, approximately half of the drugs causing liver toxicity in humans are not identified during preclinical toxicology [8].

Therefore, there is a substantial need for developing *in silico* prediction models that can accurately assess the hepatotoxic potential of new drug candidates. Such models would enable the rapid screening of large number of drugs, helping to identify those with high risks of both intrinsic and idiosyncratic DILI early in the development process. This approach not only enhances patient safety but also reduces the risk of late-stage clinical trial failures, and ultimately making drug development more efficient and cost-effective. To support the development of accurate *in silico* DILI prediction models, the US FDA has released several versions of the DILI dataset in 4-5 year intervals since 2011 [9–11]. These updates incorporate new findings, improved data quality and expand data coverage to support safer drug development and reduces the risk of late-stage clinical trial failures. Different versions of the FDA DILI datasets have been widely used in DILI prediction research community to develop Machine Learning (ML) and Deep Learning (DL) algorithms for DILI prediction .

Researchers use both traditional machine learning (ML) and deep learning (DL) models to predict drug-induced liver toxicity which poses a significant challenge in drug development [12]. In traditional ML approaches, Support Vector Machines (SVMs) have been employed to predict DILI using weighted molecular fingerprints derived from PubChem fingerprints [13]. Similarly, Random Forest (RF) models were also developed to predict mouse liver toxicity. leveraging chemical descriptors and *in vitro* ToxCast bioactivity data [14]. These models used both chemical descriptors and *in vitro* ToxCast bioactivity data, focusing on features that include both chemical and biological information [14]. Others have used ensemble learning approaches to combine predictions from multiple ML algorithms such as linear discriminant analysis, naive Bayes, SVMs, classification and regression trees, and k-nearest neighbours (kNN) [15]. These traditional ML approaches focus on integrating both chemical and biological information due to limitations in predictive performance when relying solely on data from a single domain [12].

In the recent years, deep learning (DL) models are increasingly used in the DILI prediction task pipeline, often yielding superior performance compared to traditional ML models. Below, we describe three state-of-the-art models that used the latest US FDA DILI dataset (DILIst) [11] and two state-of-the-art models that combined the FDA DILI datasets with LiverTox dataset [16], evaluated in AUC metric, which is a widely recognised standard in the DILI prediction research. The models under consideration are DeepDILI [4], Supervised Subgraph Mining (SSM) [17], DILIPredictor [18], Deep Neural Network (DNN) [19], and DNN-Graph Attention Network (DNN-GATNN) [20], each leveraging one or more advanced ML and DL techniques in the DILI prediction task pipeline.

Released in 2020, the DeepDILI model integrates conventional machine learning algorithms with a fully connected neural network to predict drug-induced liver injury. This model utilises model-level representations from each ML algorithm, rather than relying on molecular representations, to capture DILI-related information effectively. The approach includes logistic regression, kNN, SVMs, RFs, and extreme gradient boosting. The outputs from these algorithms are fed into a fully connected neural network, which serves as a meta-classifier. This neural network consists of multiple layers, using activation functions such as the hyperbolic tangent and sigmoid to transform the input into probabilities indicating the likelihood of a drug causing DILI. DeepDILI achieved 0.659 Area Under the Receiver Operating Characteristic Curve (AUC) and 0.687 Accuracy (ACC) [4].

Introduced in 2023, the Supervised Subgraph Mining (SSM) approach utilises a random walk algorithm to extract explicit subgraph features, which are then used to train a Random Forest classifier. This method iteratively generates subgraphs for DILI prediction by updating the graph transitions in a supervised manner, increasing the discrimination power of subgraph features. The SSM approach achieved an AUC of 0.691 and an ACC of 0.687, outperforming DeepDILI [17].

Published in 2024, DILIPredictor integrates predicted *in vivo* and *in vitro* data to predict DILI. The model uses nine proxy-DILI labels, such as mitochondrial toxicity and bile salt export pump inhibition, alongside chemical structural features from molecular fingerprints. DILIPredictor is the first model to combine chemical features from the FDA DILIst dataset with the predicted biological features for DILI prediction. The model achieved an AUC-PR of 0.79 and a balanced accuracy of 0.59 [18]. Although the model attained relatively low AUC-ROC of 0.63, the authors employed scaffold-based split and focused on metrics that are more relevant to safety and toxicology, such as positive likelihood ratio and precision-recall characteristics to emphasise correct identification of true toxic compounds in the real-world drug discovery process.

Other studies in DILI prediction task have combined the FDA-released DILI datasets with an additional DILI dataset; LiverTox [16].

Reported in 2024, Yang et al. introduced a fully connected deep neural network (DNN) trained on different types of molecular fingerprints, including substructure-based [21, 22], topological [23, 24], and circular fingerprints [25]. The best-performing DNN used the circular fingerprints (ECFP6), achieving an AUC of 0.713 [19].

Building upon the work of Yang et al., Wibowo et al. proposed an ensemble model combining a DNN with a graph attention neural network (DNN-GATNN) in 2025. This approach utilised both substructure-based fingerprint [21] and graph-structured data, allowing model to effectively capture local substructures as well as molecular graph information. The ensemble model achieved an AUC of 0.757, outperforming previous state-of-the-art models and demonstrating the advantage of incorporating graph-based representations in the DILI prediction task [20].

Graph neural networks (GNNs) have emerged as a powerful class of DL models capable of processing graph-structured data, making them particularly suitable for molecular prediction tasks. Unlike the previous models that rely on predefined chemical descriptors such as chemical fingerprints or molecular physicochemical parameters, GNNs can directly learn from the structural information encoded within molecular graphs. This ability to capture complex relationships and local structural features has allowed their applications in various molecular property prediction tasks in cheminformatics research.

Graph Convolutional Network (GCN) [26] has been effectively employed in Amgen’s absorption, distribution, metabolism, excretion (ADME) prediction service. By learning directly from molecular graphs instead of relying on traditional chemical descriptor values, GCN significantly improved prediction accuracy [27].

In drug-target binding affinity studies, Graph Attention Network (GAT) [28] enhanced the prediction performance by using distance-aware attention mechanisms to incorporate spatial information between drug-target binding pocket [29].

Toxicity prediction for drugs has been improved through a hybrid approach where Graph Sample and Aggregation (GraphSAGE) [30] was used to extract structural features from molecular graphs, while Bi-directional Gated Recurrent Unit (BiGRU) leveraged SMILES strings to provide contextual information [31].

In the study of drug-drug interactions, the combined application of Graph Isomorphism Network (GIN) [32] and Node2Vec [33] facilitated the extraction of isomorphic molecular graph features, proving effective in improving predictions for structurally similar drugs [34].

Our primary objective is to explore the application of GNNs in the DILI prediction task while leveraging their ability to learn directly from our custom-designed molecular graph representations. Our research is motivated by several key observations.

A thorough review of the existing literature reveals that no current models exclusively utilise GNN as a stand-alone method in DILI prediction. While GNNs have demonstrated success in other molecular property prediction tasks, their potential in DILI prediction remains unexplored. This presents an opportunity to investigate whether the GNNs can enhance the predictive performance of DILI models.

Among the recent state-of-the-art models, DNN-GATNN stands out as the method that predicts DILI using solely chemical representation. This approach is important because models that incorporate additional features such as *in vivo* or *in vitro* data require extensive experimental setups and expert interpretation, increasing the complexity and cost down the drug development pipeline.

Additionally, we will validate the versatility and generalisability of our GNN models by testing them on other molecular property prediction tasks using the ClinTox, Blood-Brain Barrier Permeability (BBBP), and Beta-Secretase 1 (BACE) datasets. This broader evaluation aims to assess whether our GNN models that perform well in DILI prediction can also demonstrate improved predictive accuracy across other molecular property prediction tasks.

For these molecular prediction tasks, several methods have demonstrated state-of-the-art performance by utilising different types of data in conjunction with different architectures. Graph topology induced optimal transport (GTOT-tuning) [35] pretrained a GIN using only a single molecular graph (as we are using here) followed by fine-tuning for specific molecular property prediction tasks. Other methods use additional data such as molecular graphs with additional 200 chemical descriptors, including extended-connectivity fingerprint (ECFP) [25], in a directed message passing neural network (D-MPNN) [36], or by geometric graph embedding of two molecular fingerprints (ECFP and using MACCS keys [21]) using geometry-enhanced molecular representation learning (GEM) [37]. More recently, graph embeddings generated from GIN and GAT models with mixed molecular fingerprints were used in a dual-graph contrastive learning (DGCL) approach [38], and by integrating ECFP with embeddings from a graph-based model [39] and the MoLFormer language model [40, 41].

Given these insights, this study aims to evaluate the effectiveness of the most prominent GNN architectures: GCN, GAT, GraphSAGE and GIN, in DILI and the other molecular prediction tasks using only chemical representations as input. By focusing solely on the molecular graph representations, we seek to assess the capability of these GNNs capturing relevant structural information and improve DILI and the other molecular property prediction performance compared to the recent state-of-the-art models.

## Data and methods

### Drug induced liver injury dataset

The most recent release of the drug-induced liver injury (DILI) dataset was downloaded from the US FDA official website (accessed on 28/06/2024). The dataset is the largest binary DILI annotation dataset available which composes of 768 DILI positive and 511 DILI negative drugs classified by Thakkar et al. [11]. The classification was performed by augmenting the DILIrank dataset [10] with data from four large literature datasets: LiverTox [42], Suzuki [43], Greene [44], and Zhu [45]. This involved a concordance analysis and incremental augmentation process, ensuring consistency with the DILIrank classifications. The augmentation process only included drugs that met a 75% concordance criterion for their respective categories: DILI positive or negative [11]. The dataset contains compound names and binary labels of drugs, 1: DILI positive and 0: DILI negative. SMILES strings were retrieved from the PubChem database using the requests library (version 2.31) in Python (version 3.10).

### Other molecular property prediction datasets

MoleculeNet is a comprehensive benchmark suite for machine learning tasks in molecular property prediction [46]. This benchmark dataset is curated from public datasets and provides standardised metrics to assess model performance. We utilised three datasets from MoleculeNet to evaluate our model performance beyond the DILI dataset. The ClinTox dataset includes 1491 drug molecules and features two classification tasks aimed at evaluating model performance on FDA approval and general toxicity. The Blood–Brain Barrier Penetration (BBBP) dataset is derived from research focused on predicting the permeability of compounds through the blood-brain barrier, which is crucial for developing drugs targeting the central nervous system. It contains binary labels for over 2,053 compounds indicating their permeability properties. The BACE dataset provides both quantitative (IC50) and qualitative (binary classification) data related to inhibition of the enzyme beta-secretase 1 (BACE-1), which is implicated in Alzheimer’s disease. It consists of 1,522 compounds, and we utilised the qualitative data for our analysis. We obtained the chemical structures for these datasets using the provided SMILES strings. The dataset was downloaded using PyTorch Geometric (version 2.5.3, accessed on 15/04/2024) [47].

### Drug data pre-processing

We created an in-house molecule pre-processing and standardisation algorithm to ensure that molecular representations in the DILI dataset is cleaned, normalised and validated for further analysis and use in cheminformatics applications. The goal of this process is to convert potentially diverse and non-standardised SMILES strings into a consistent format, which is crucial for reliable downstream analysis such as structure toxicity relationship studies including current study. Throughout the pre-processing steps, drug molecules that could not be pre-processed were recorded with appropriate error messages and saved. The data used for pre-processing is available in the file DILIst_standardised.csv in the supporting documents.

#### Chemical element screening

The process begins by iterating through each SMILES string in the DILI dataset. The algorithm attempts to convert these strings into molecular objects in RDKit mol formats (RDKit version 2024.03.1). For valid molecules, the algorithm iterates through the chemical elements of each molecule to ensure that the molecule only contains 10 common elements typically used in organic chemistry and drug synthesis. These elements include Hydrogen, Carbon, Nitrogen, Oxygen, Fluorine, Phosphorus, Sulphur, Chlorine, Bromine and Iodine, and these elements are commonly found in stable, drug-like molecules with the desired pharmacokinetic and pharmacodynamic properties. This step is vital for filtering out drugs with uncommon elements which are less frequent in FDA-approved drugs [48], thereby excluding specialised drugs with uncommon elements (such as Acetarsol with Arsenic, and Bortezomib with Boron) which may cause bias in training the DILI prediction models. We have excluded 34 Drugs with the uncommon elements which can be found in our GitHub.

#### Molecule standardisation

Once a molecule passes the chemical element screening, the molecular standardisation process begins. This involves molecular normalisation steps such as removing explicit hydrogen atoms, disconnecting metal atoms, and neutralising ionic charges to produce a standardised molecular representation. A critical part of this process is identifying the parent molecule, especially in structures with multiple fragments, which is often encountered in medicinal chemistry. The parent molecule is the largest fragment, and its identification focuses the analysis on the active structure of interest, allowing smaller, less relevant fragments such as salts and solvents to be ignored. After normalisation and identification of the parent molecule, the algorithm enumerates all possible tautomers to standardise tautomeric forms. This ensures consistency across different tautomeric states of a molecule by identifying a single tautomeric form. This step is particularly important for compounds that can exist in multiple tautomeric forms, such as Warfarin [49].

### Molecular graph generation

#### Molecule optimisation

The SMILES string of each drug was used to generate a 2D molecular structure in RDKit mol format, after which hydrogen atoms were added to ensure each structure has all its hydrogen atoms explicitly represented which is essential in molecular simulations and optimisations. Then, An initial 3D conformation of the drug molecule was generated using distance geometry methods as implemented in RDKit’s EmbedMolecule function [23]. The generation process involved creating a spatial arrangement of atoms based on distance constraints derived from the chemical bonds, angles, and torsions by its molecular structure. During the 3D conformation generation, a maximum of 5,000 attempts was allowed, starting from random atomic coordinates to produce a valid structure that satisfied all geometric constraints [50]. The conformer generation only ensures that generated conformation is reasonably plausible and follows the basic geometric constraints, therefore, we further optimised the conformer using Merck Molecular Force Field (MMFF) method, as implemented in RDKit’s MMFFOptimizeMolecule function [23]. MMFF takes various intra-molecular forces into account including bond stretching, angle bending, torsions, van der Waals interactions, and electrostatics, to generate molecular geometries that are consistent with experimental data by adjusting the positions of atoms to minimise the overall molecular energy to find the most stable conformation [51].

#### Creation of custom graph dataset

**Table 1:** Comparison of node and edge features included in the PyTorch Geometric MoleculeNet dataset and our custom graph dataset

Feature type	PyTorch geometric moleculeNet	Our custom graphs
Node features		
Atomic number	$\checkmark$	$\checkmark$
Atom degree	$\checkmark$	$\checkmark$
Formal charge	$\checkmark$	$\checkmark$
Hybridisation	$\checkmark$	$\checkmark$
Aromaticity	$\checkmark$	$\checkmark$
Number of hydrogens	$\checkmark$	$\checkmark$
In ring	$\checkmark$	$\checkmark$
Chirality	$\checkmark$	$\checkmark$
Radical electrons		$\checkmark$
Gasteiger partial charges		$\checkmark$
Total valence		$\checkmark$
Explicit valence		$\checkmark$
Edge features		
Bond type	$\checkmark$	$\checkmark$
In ring	$\checkmark$	$\checkmark$
Bond length		$\checkmark$

The custom graph dataset incorporates additional features such as Gasteiger partial charges, total valence, and bond length derived from 3D optimised conformers

The PyTorch Geometric MoleculeNet datasets are widely used for machine learning tasks in molecular property prediction. These datasets represent molecules as graphs, where nodes correspond to atoms and edges represent bonds. Node features typically include atomic number, degree, hybridisation, and aromaticity [47]. PyTorch MoleculeNet datasets [46] are commonly used in graph-based algorithms, such as graph neural networks, which utilise these structural representations to analyse interactions between atoms for molecular property prediction tasks. However, these datasets do not include 3D conformational data, which limits their ability to capture spatial molecular features directly.

In contrast, our custom graph dataset (Table 1) incorporates molecular optimisation, providing 3D spatial information for a more accurate depiction of molecular geometry, from which bond lengths are calculated as edge features using RDKit’s rdMolTransforms.GetBondLength function [23], which computes the Euclidean distance between bonded atoms based on their 3D coordinates. While these bond lengths do not represent the true molecular conformation in a biological environment, this approach offers a more realistic approximation close to experimental data, rather than relying on average bond lengths from empirical tables [52, 53]. This approach is a standard procedure in the field of medicinal chemistry for molecular docking simulations where accurate geometry is crucial as it influences molecular stability within ligand-target binding sites [54], but it has not been widely adopted in the AI community.

Furthermore, our custom dataset created in this study adds features such as Gasteiger partial charges [55], total valence, and explicit valence. Gasteiger charges were computed using RDKit’s ComputeGasteigerCharges function, while valence properties were obtained via RDKit’s atom object methods GetTotalValence and GetExplicitValence [23]. These additional features provide detailed information about the electronic distributions within the molecule supporting more accurate predictions of complex molecular properties by offering a more detailed representation of molecular structure [56].

#### Bond length change analysis and statistical testing

The bond length changes were evaluated as a percentage difference before and after molecular optimisation for each molecule. To summarise the data, we computed basic statistical metrics, including the mean, median, standard deviation, and range, for both standardised and non-standardised SMILES representations using the describe function from the Pandas library (version 2.2.2) [57]. To statistically assess the differences in bond length changes between standardised and non-standardised SMILES, we employed a paired t-test. This test is suitable for comparing two related samples, specifically the bond length changes for the same set of molecules before and after standardisation. The paired t-test was conducted using the ttest_rel function from the from Scikit-learn library (version 1.3.1) [58]. This approach allows us to determine whether the standardisation process results in statistically significant differences in bond length changes.

#### Creation of basic molecular graph representation

In our feature comparison study, we utilised a basic molecular graph representation as a foundational approach to model molecular structures. This representation simplifies each molecule into a graph, where atoms are depicted as nodes and bonds between them as edges. In this basic graph representation, the primary features include the atomic number for nodes and the bond type for edges. These features provide basic information about the molecular structure, capturing fundamental aspects necessary for initial property prediction tasks. Many graph-based prediction approaches such as the SSM model utilised this basic molecular graph as input features [17].

### Training and evaluation of deep graph neural networks

In this study, we developed deep graph neural networks (GNNs) to perform graph-based learning tasks using our custom-designed graph dataset, which includes node features, edge features, and target labels for the binary classification of drug-induced liver injury (DILI). The models used in this study include Graph Convolutional Networks (GCNs), Graph Attention Networks (GATs), GraphSAGE, and Graph Isomorphism Networks (GINs). These architectures were chosen to accurately capture the relationships within molecular graphs, thereby improving the predictive accuracy of molecular property prediction. To ensure robust training, optimisation and evaluation of these models, we employed a nested cross-validation (CV) framework for hyperparameter optimisation, model reinitialisation and sequential warm starts strategies. These methods aimed to maximise performance stability across different random initialisations and improve the generalisability of our models. Additionally, we conducted the same experiment on other binary molecular property prediction datasets, including Clintox, BBBP, and BACE, sourced from the MoleculeNet dataset [46] to evaluate our model performance across different molecular property prediction tasks and validate the effectiveness of our training approach. The subsequent sections provide a detailed account of the experimental setup, including model architectures, training procedures, and the evaluation metrics used to measure model performance in different stages of our nested CV framework.

**Fig. 1:**
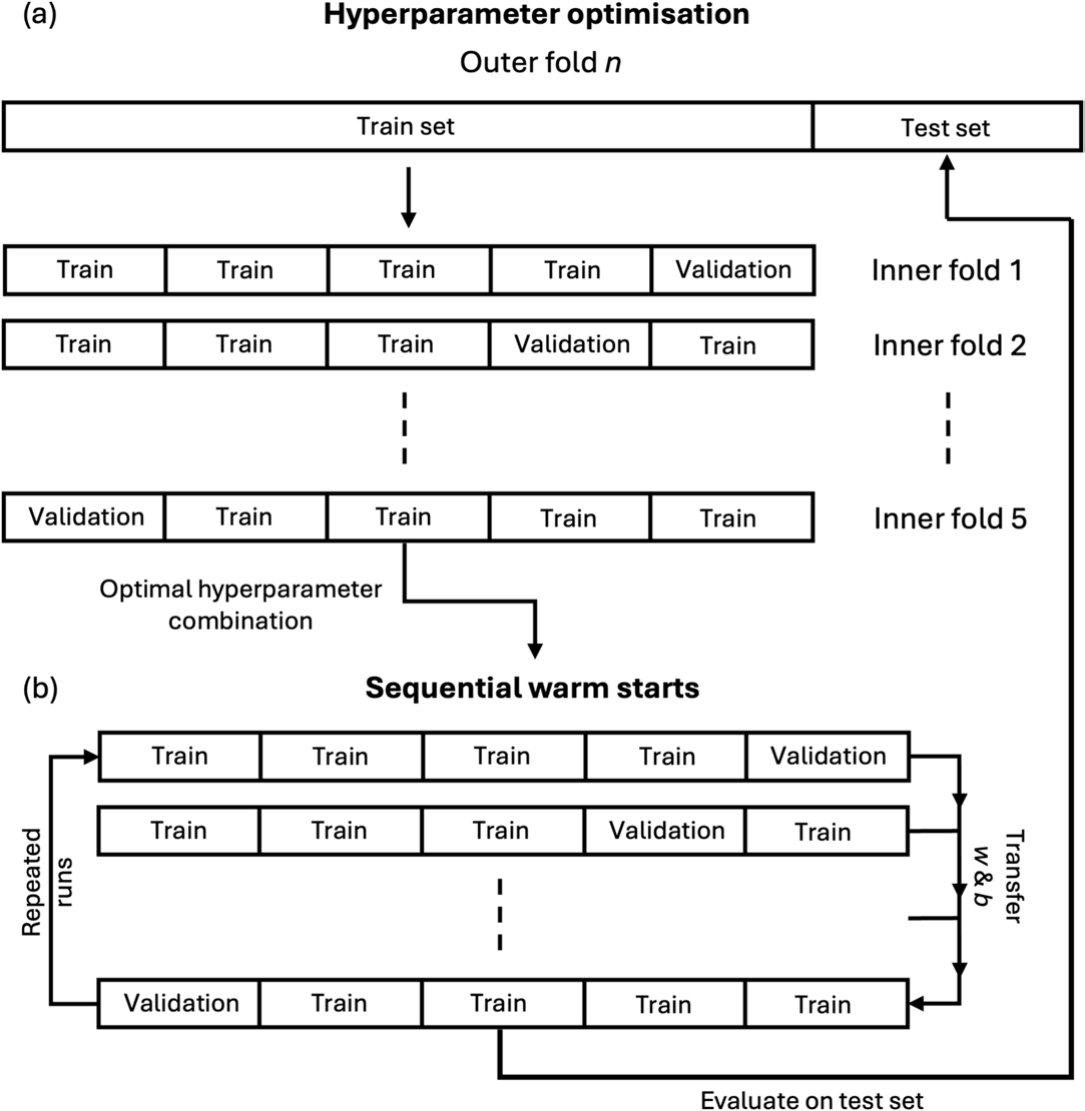
Nested Cross-Validation Framework for Hyperparameter Optimisation and Sequential Warm Starts. This figure presents a workflow of hyperparameter optimisation, sequential warm starts for model training and evaluation in the nested cross-validation framework. **a** Hyperparameter Optimisation to find the optimal hyperparameter across five inner folds. **b** Sequential warm starts for fine-tuning the model parameters within the inner loop for multiple runs. Subsequently, the trained model was evaluated on the outer fold test set

#### Nested cross-validation for hyperparameter optimisation

Figure 1a shows the nested CV framework for outer fold hyperparameter optimisation. For each GNN model, the data was initially divided into four stratified outer folds. In each iteration of the outer folds, one fold served as the test set (25% of the data), while the remaining three folds were further split into five stratified folds for the inner loop for hyperparameter tuning. This process of splitting was used to ensure the test splits used for evaluation (see section below on metrics) of the models with hyperparameters optimised remain independent. Moreover, performing CV in the inner loop allows the optimal hyperparameters to be found using a validation split and prevents overfitting (thereby making it more generalisable). In each iteration of this inner loop, one fold was used as the validation set (15%) for hyperparameter optimisation and the remaining four folds were used as the inner fold training set (60%). The model was trained and evaluated using 135 hyperparameter combinations including batch size, number of hidden channels, dropout rate and learning rate, listed in Supplementary Table 1. The optimal hyperparameter combination for each outer fold was selected based on a combined ranking of the highest median and minimum validation AUC scores across the inner folds. This approach ensured that the selected hyperparameters performed consistently well across different data splits. This entire process was repeated across all outer folds (n=4). For data splitting we used stratified cross-validation algorithm from scikit-learn (version 1.3.1).

#### Model reinitialisation and sequential warm starts for fine tuning

After identifying the optimal hyperparameters, we addressed the variability introduced by random weight initialisation by training models with 20 different random seeds from PyTorch (version 2.3.1) [59]. For each outer fold, we selected the best-performing seed based on the highest combined rank of median and minimum validation AUC scores obtained from the inner folds. This model reinitialisation step aimed to enhance performance stability across all inner folds and ensured the reproducibility for the further work. To further refine the models, we employed a sequential warm starts method for fine-tuning the parameters (see Fig. 1b). Each run in the sequential warm starts method consisted of a complete cycle of training the model across all inner folds without reinitialising the parameters, allowing the model to incrementally refine its parameters by learning from different subsets of the data. Specifically, the model is first trained on inner fold 1. It then continues training on inner fold 2 using the learned parameters from fold 1, and this process repeats through folds 3, 4, and 5. Each run yielded five validation AUC scores per run per outer fold, resulting in a total of 20 validation AUC scores per run across all four outer folds. We repeated the sequential warm starts process for at least three runs to assess whether continued training led to significant improvements. Upon the completion of each run, we conducted significance testing using the Wilcoxon rank sum test to compare the current run’s AUC scores with those from the previous two runs [60]. We applied a significance level of alpha = 0.05, which was then adjusted using the Sidak correction for two comparisons to control the family-wise error rate [61]. If no statistically significant improvement was observed (i.e. both p-values exceeded the corrected alpha), we halted the sequential warm starts for that model. Throughout the training process, we applied an early stopping function that monitored the validation AUC scores and halted training for a fold if no improvement was observed over 50 epochs. The statistical significance testing and the early stopping prevented further training beyond the point of meaningful improvement and model overfitting to a certain inner fold validation set to enhance generalisability of the GNN models.

### Model architecture

The experimental setup involved implementing and evaluating four Graph Neural Network (GNN) architectures: Graph Convolutional Network (GCN), Graph Attention Network (GAT), GraphSAGE, and Graph Isomorphism Network (GIN). Each architecture was optimised for the dataset using a hyperparameter grid search within the inner CV loop and tested on the unseen test dataset in the outer CV loop within the first part of the hyperparameter optimisation (Fig. 1a). The following equations define the convolutional layers we implemented from PyTorch geometric (version 2.5.3).

GCN aggregates information from a node’s neighbours using normalised adjacency matrix. In equation 1, $\textbf{h}_i^{(l)}$ is the embedding of node *i* at layer *l*, $\mathcal {N}(i)$ denotes the set of neighbours of node *i*, and $\hat{d}_i = 1 + \sum _{j \in \mathcal {N}(i)} e_{j,i}$ is the degree of node *i* including self-loops, where $e_{j,i}$ signifies the edge weight of nodes j and i. $\textbf{W}^{(l)}$ is the trainable weight matrix for layer *l*, and $\sigma$ is the activation function:

1 $$\begin{aligned} \textbf{h}_i^{(l+1)} = \sigma \left( \sum _{j \in \mathcal {N}(i) \cup \{i\}} \frac{1}{\sqrt{\hat{d}_i \hat{d}_j}} \textbf{W}^{(l)} \textbf{h}_j^{(l)} \right) \end{aligned}$$

GAT introduces attention mechanisms to weight the contributions of neighbouring nodes. In equation 2 below, $\alpha _{ij}^{(l)}$ is the attention coefficient between nodes *i* and *j* that replaces the degree normalisation in equation 1:

2 $$\begin{aligned} \textbf{h}_i^{(l+1)} = \sigma \left( \sum _{j \in \mathcal {N}(i) \cup \{i\}} \alpha _{ij}^{(l)} \textbf{W}^{(l)} \textbf{h}_j^{(l)} \right) \end{aligned}$$

The attention coefficient from equation 2 is computed (and normalised) using a LeakyReLU activation function in equation 3 below, where $\textbf{a}^{(l)}$ is a learnable attention vector and || denotes concatenation:

3 $$\begin{aligned} \alpha _{ij}^{(l)} = \frac{\exp \left( \text {LeakyReLU} \left( \textbf{a}^{(l)^T} \left[ \textbf{W}^{(l)} \textbf{h}_i^{(l)} \, || \, \textbf{W}^{(l)} \textbf{h}_j^{(l)} \right] \right) \right) }{\sum _{k \in \mathcal {N}(i) \cup \{i\}} \exp \left( \text {LeakyReLU} \left( \textbf{a}^{(l)^T} \left[ \textbf{W}^{(l)} \textbf{h}_i^{(l)} \, || \, \textbf{W}^{(l)} \textbf{h}_k^{(l)} \right] \right) \right) } \end{aligned}$$

GraphSAGE updates node embeddings by aggregating information from neighbouring nodes using a mean aggregation function. In equation 4, $\textbf{W}_1^{(l)}$ and $\textbf{W}_2^{(l)}$ are trainable weight matrices at layer *l*, and the mean aggregation ensures that the contributions from neighbours are normalised by the number of neighbours of node i:

4 $$\begin{aligned} \textbf{h}_i^{(l+1)} = \sigma \left( \textbf{W}_1^{(l)} \textbf{h}_i^{(l)} + \textbf{W}_2^{(l)} \cdot \frac{1}{|\mathcal {N}(i)|} \sum _{j \in \mathcal {N}(i)} \textbf{h}_j^{(l)} \right) \end{aligned}$$

Finally, for updating weights, GIN employs sum aggregation followed by a trainable multilayer perceptron (MLP) to improve expressive capacity of node representations. In equation 5, $\epsilon$ is a fixed scalar parameter, and $\text {MLP}$ represents an MLP applied to the aggregated features:

5 $$\begin{aligned} \textbf{h}_i^{(l+1)} = \text {MLP} \left( (1 + \epsilon ) \cdot \textbf{h}_i^{(l)} + \sum _{j \in \mathcal {N}(i)} \textbf{h}_j^{(l)} \right) \end{aligned}$$

#### Generalised GNN model

The generalised GNN model class utilised a modular architecture that allows for the specification of different convolutional layer types. We implemented the following convolutional types: GCNConv, GATConv, and SAGEConv. The model comprised three convolutional layers, each followed by a ReLU activation function. The architecture is detailed below:

- **Weight Initialisation:** Xavier initialisation was applied to all network weights to enhance convergence [62].
- **Convolutional Layers:**
  - **Layer 1:** Convolutional layer with output dimensions of hidden_channels*2, followed by ReLU activation.
  - **Layer 2:** Convolutional layer with output dimensions of hidden_channels, followed by ReLU activation.
  - **Layer 3:** Convolutional layer with output dimensions of hidden_channels/2, followed by ReLU activation.
- **Dropout Layer:** A dropout rate was employed after the final convolutional layer to mitigate overfitting.
- **Global Pooling:** Global mean pooling was applied to aggregate node features into a fixed-size graph-level representation.
- **Output Layer:** A linear layer was used to map the pooled representation to a single output logit. The sigmoid activation function was incorporated during the loss computation using BCEWithLogitsLoss or CrossEntropyLoss for the ClinTox classification task.

#### Graph isomorphism network (GIN) model

The GIN model featured a specialised architecture leveraging GINConv layers. Each GINConv layer employed a multi-layer perceptron (MLP) for feature transformation. The model architecture is summarised as follows:

- **Weight Initialisation:** Xavier initialisation was applied to all weights to promote stable training.
- **GIN Convolutional Layers:**
  - **Layer 1:** A GINConv layer with an MLP comprising two linear layers and ReLU activation, transforming features to hidden_channels*2.
  - **Layer 2:** A GINConv layer with an MLP that transforms features to hidden_channels.
  - **Layer 3:** A GINConv layer with an MLP reducing the feature size to hidden_channels/2.
- **Dropout Layer:** A dropout rate was employed after the final GIN convolutional layer to reduce overfitting.
- **Global Pooling:** Global mean pooling was applied to derive a fixed-size graph-level representation.
- **Output Layer:** A linear layer mapped the pooled representation to the final output logit, with the sigmoid activation function handled by the BCEWithLogitsLoss or CrossEntropyLoss for the ClinTox classification task.

### Evaluation metrics

Following the sequential warm starts, the models were evaluated on the test set of each outer fold using several metrics. Accuracy measured the proportion of correctly classified instances. The F1-score, calculated as the harmonic mean of precision and recall, assessed the balance between precision and sensitivity. The Area-Under-the-ROC-Curve (AUC) evaluated the model’s ability to distinguish between classes, while Matthew’s Correlation Coefficient (MCC) provided a balanced assessment by accounting for true and false positives and negatives. While median, maximum, and minimum values might be more suitable for a lower number of evaluation metrics to better capture variability, we chose mean and standard deviation values, as other state-of-the-art models reported these values [4, 17, 18]. To allow comparison of our results to prior as well as future work on the same datasets, we report results on all metrics for the test set (split from the remainder of the data as first step, Fig. 1). AUC is commonly used by others to show the performance of binary classifiers, however it is not suitable for imbalanced datasets (whereas F1-score and MCC are more suitable for reporting on datasets with class imbalances). We report the AUC to allow comparison of our results to work only reporting AUC. The Area Under the Precision-Recall Curve (AUC-PR) is not reported here as this is more suited for datasets where the positive class is rare opposed to the imbalance seen in the datasets we demonstrate the method on.

## Results

### Processed US FDA DILI dataset and comparison with previous studies

There are 1,279 drugs in the latest release of the FDA DILI dataset [11]. We were unable to retrieve SMILES strings for 33 DILI positive and 45 DILI negative drugs using the request method. The failure to retrieve these strings is primarily due to the drugs being either large biomolecule drugs (e.g., Gemtuzumab ozogamicin, Secretin) or involved in metal replacement therapy (e.g., Ferumoxytol), or due to having different structural variations. Then 1,201 drugs went through preprocessing and molecular standardisation. Following these steps, 1,167 drugs remained and all of these passed the molecular optimisation process during the graph generation step.

**Table 2:** Comparison of the number of drugs and the positive-to-negative (+ to −) ratios in different DILI studies

Studies	DILI drugs	+ to − ratio
US FDA DILIst [11]	1,279	1.50:1
Our data	1,167	1.63:1
DILIPredictor [18]	1,111	1.81:1
DeepDILI [4]; SSM [17]	1,002	1.52:1

The table highlights the differences in dataset sizes and class balance across different DILI studies. The studies include the original US FDA DILI dataset, our processed dataset, DILIPredictor, DeepDILI, and SSM. Our dataset retains a greater number of drugs from the original FDA dataset, with a positive-to-negative ratio closer to the original, indicating a more balanced dataset compared to the DILIPredictor study

Our data processing strategies have yielded more drugs from the FDA DILI dataset compared to previous studies that used FDA-released DILI datasets (Table 2). The latest FDA DILI dataset is called DILIst [11], which contains 1,279 drugs with a positive to negative ratio of 1.50:1. DeepDILI used 1,002 drugs after excluding biologics, drug mixtures, organometallics, and inorganics [4], and SSM used the same dataset from the DeepDILI study [17]. DILIPredictor improved upon this by combining the latest (DILIst [11]) and the previous version (DILIRank [10]) of the FDA DILI dataset. After applying their molecular standardisation method, DILIPredictor dataset includes 1,111 drugs with a more imbalanced positive to negative ratio of 1.81:1 compared to the original dataset [18]. In contrast, our pre-processing and molecular standardisation strategies resulted in 1,167 drugs, leveraging more information from the FDA DILI dataset and achieving a positive to negative ratio of 1.63:1. This indicates that our dataset is closer to the balance seen in the original dataset compared to the DILIPredictor dataset. We identified that 8 drugs from the DeepDILI study and 122 drugs (including 37 drugs from DILIRank) from the DILIPredictor study are not present in our dataset. These drugs, along with those for which SMILES retrieval failed, are available in our GitHub. The DNN [19] and DNN-GATNN [20] studies combined LiverTox dataset with the FDA DILI dataset; therefore, they were not included in this study.

Furthermore, to assess the effects of our molecular standardisation method on molecular bond length changes following molecular optimisation, we computed statistical metrics (mean, median, standard deviation) of bond length changes for both standardised and non-standardised molecules, as shown in Supplementary Table 2. We observed that our molecular standardisation method reduces extreme outliers in maximum bond length changes from 14.44% to 7.45% and standard deviation decreasing from 0.62% to 0.53%. Although the difference in mean bond length change between standardised and non-standardised molecules is not statistically significant (p=0.265), applying the molecular standardisation method is beneficial for ensuring data quality and reproducibility in computational chemistry [63]. The data used for this analysis, including bond lengths after optimisation of drug molecules, is available in our GitHub.

### Model performance in the DILI prediction task

**Table 3:** Performance Metrics (mean ± std) of GNN Models on the DILI Prediction Task

Metric	GCN	GAT	GIN	GraphSAGE
Validation AUC				
Hyperparameter optimisation	**0**.**627** ± 0.034	**0**.**627** ± 0.031	0.626 ± 0.032	0.624 ± 0.032
Model reinitialisation	0.670 ± 0.037	**0**.**695** ± 0.047	0.671 ± 0.033	0.692 ± 0.043
Sequential warm starts	0.881 ± 0.062	0.728 ± 0.054	0.916 ± 0.051	**0**.**953** ± 0.061
Test AUC	0.847 ± 0.058	0.728 ± 0.059	0.874 ± 0.027	**0**.**897** ± 0.043
Test accuracy	0.733 ± 0.081	0.619 ± 0.049	0.798 ± 0.058	**0**.**836** ± 0.095
Test F1-score	0.735 ± 0.098	0.611 ± 0.069	0.814 ± 0.062	**0**.**846** ± 0.106
Test MCC	0.533 ± 0.124	0.323 ± 0.078	0.628 ± 0.094	**0**.**698** ± 0.148

The table highlights validation AUC scores at different stages of the Nested Cross-Validation (CV) framework and test performance metrics for GCN, GAT, GIN, and GraphSAGE models. Validation AUCs are reported for hyperparameter optimisation, model reinitialisation, and after applying the Sequential Warm Starts method. These steps build upon each other, with the optimal hyperparameters used in the model reinitialisation and the best models subsequently used for the sequential warm starts to progressively enhance the model performance. The test metrics include AUC, accuracy, F1-score, and MCC, reported as mean ± standard deviation. Our method improved performance by leveraging progressive learning on training subsets and statistical significance testing to optimise generalisation. The best mean values in each metric are highlighted in bold

Table 3 summarises the performance of the four GNN models on the DILI prediction task across different stages of our Nested Cross-Validation (CV) framework, along with their test performance metrics. After hyperparameter optimisation, all models demonstrated comparable initial performance with mean validation AUC scores ranging between 0.624 and 0.627. During model reinitialisation, performance improved for all models, with increases in mean validation AUC ranging from 0.043 to 0.068. The largest improvements were observed for GAT and GraphSAGE.

Further refinement through the sequential warm starts method produced the highest performance improvements. GraphSAGE achieved the best validation performance, with a mean AUC of 0.953, followed by GIN (0.916) and GCN (0.881). GAT showed a modest improvement, reaching 0.728. This discrepancy in improvement is likely influenced by the different numbers of repeated runs performed for each model (GCN: 4, GAT: 1, GraphSAGE: 3, and GIN: 5, see Supplementary Figure 1). Specifically, GAT’s sequential warm starts halted earlier than the other models since statistical significance testing concluded that there was no further significant improvement.

On the test sets, GraphSAGE achieved the highest performance across all evaluation metrics, including test AUC (0.897 ± 0.043), accuracy (0.836 ± 0.095), F1-score (0.846 ± 0.106), and MCC (0.698 ± 0.148), followed by the GIN model. The variation in test performance among the models aligns with the trends observed in their validation AUC scores obtained during the sequential warm starts method. These results indicate that the sequential warm starts method effectively enhances model performance by leveraging progressive learning on training subsets and statistical significance testing to optimise generalisation.

### Model performance in other prediction tasks: basic features vs our custom features

**Table 4:** Comparison of test AUC scores (mean ± std) for GNN models using basic vs. custom features across four datasets

Dataset	Feature Set	GCN	GAT	GIN	GraphSAGE
DILI	Basic	0.537 ± 0.022	0.551 ± 0.022	0.511 ± 0.028	0.723 ± 0.045
	Custom	0.847 ± 0.058	0.728 ± 0.059	0.874 ± 0.027	**0**.**897** ± 0.043
ClinTox	Basic	0.502 ± 0.072	0.486 ± 0.071	0.462 ± 0.054	0.723 ± 0.045
	Custom	**0**.**918** ± 0.028	0.901 ± 0.043	0.897 ± 0.055	0.871 ± 0.051
BBBP	Basic	0.500 ± 0.101	0.358 ± 0.028	0.517 ± 0.074	0.871 ± 0.019
	Custom	0.990 ± 0.006	0.973 ± 0.020	0.981 ± 0.012	**0**.**993** ± 0.006
BACE	Basic	0.515 ± 0.061	0.541 ± 0.083	0.518 ± 0.040	0.604 ± 0.031
	Custom	0.906 ± 0.053	0.845 ± 0.047	0.620 ± 0.066	**0**.**953** ± 0.020

This table presents the test AUC scores for each GNN on four different datasets: DILI, ClinTox, BBBP, and BACE. For each model and dataset, results are shown separately for basic molecular graph features (atomic number and bond type) and our custom-designed features, which include an enriched set of chemical and structural information (see Table 1). The highest AUC scores for each dataset are highlighted in bold

Table 4 compares the test AUC scores of our GNN models using basic chemical graphs versus our custom-designed molecular graphs across four datasets: DILI, ClinTox, BBBP, and BACE. This analysis evaluates the effectiveness of chemical feature engineering on model performance in different prediction tasks, including toxicity (DILI and ClinTox), permeability (BBBP), and biochemical activity (BACE).

In the basic feature set, which utilises only atomic number and bond type as node and edge features, the models generally exhibited low AUC scores close to 0.5, indicating limited predictive performance. Specifically, GCN, GAT, and GIN showed minimal performance, with AUCs ranging from approximately 0.358 to 0.551 across the datasets. An exception was observed with GraphSAGE, which achieved comparatively higher AUC scores of 0.723 on the DILI dataset, 0.739 on ClinTox, and 0.871 on BBBP. This improved performance can be attributed to GraphSAGE’s inductive learning approach, which effectively aggregates information from neighbouring nodes to capture structural patterns and relationships within the graphs, partially compensating for the limited features in the basic molecular graphs. However, on the BACE dataset, even GraphSAGE’s performance was modest, with an AUC of 0.604, suggesting that the basic features were insufficient for distinguishing active compounds in this biochemical activity prediction task.

In contrast, the use of the custom-designed features, which include a richer set of chemical and structural information, resulted in significant improvements in AUC values across all datasets. For the DILI dataset, GIN showed the highest improvement, with an AUC increase from 0.511 using basic features to 0.874 with custom features, reflecting an improvement of 0.363. For the Clintox dataset, GCN displayed the most notable improvement, with the AUC improving from 0.502 with basic features to 0.918 with custom features, an increase of 0.416. For the BBBP dataset, GCN showed the greatest improvement in AUC values, improving from 0.500 with basic features to 0.990 with custom features, marking a substantial increase of 0.490. For the BACE dataset, GraphSAGE achieved the highest improvement, with AUC values rising from 0.604 in the basic feature set to 0.953 with the custom features, with an improvement of 0.349.

Overall, GraphSAGE was the best-performing model in the DILI, BBBP, and BACE prediction tasks, while GCN achieved the highest performance in the ClinTox prediction task. These best-performing models will be compared to the state-of-the-art graph classification models in the discussion section.

## Discussion

### Impact of the nested CV framework on model performance

The nested CV framework employed in this study significantly improved the performance of our GNN models by systematically selecting optimal hyperparameter combinations, reducing variability from random weight initialisation, and fine-tuning the model parameters with sequential warm starts on different subsets of the data within the inner CV loop. In the hyperparameter optimisation stage, we selected the optimal hyperparameter combination by evaluating on all five inner folds using both median and minimum validation AUC scores. This approach ensured that the selected hyperparameter combination was not overly tuned to a specific inner fold but generalised well across diverse data splits therefore minimising the likelihood of selecting sub-optimal hyperparameter combinations (see Supplementary Figure 2). In our preliminary investigation of the model reinitialization method, we ran 10-fold CV with 5 different seeds and observed performance discrepancies due to random weight initialization (see Supplementary Figure 3). This outcome is particularly relevant in deep learning models that are sensitive to local minima where variations in initial weights and biases can lead to differences in performance outcome. To address this variability, we trained the models with 20 different seeds in the main study. For each outer fold, the best-performing seed was selected based on a combined ranking of the median and minimum validation AUC scores across all five inner folds (see Supplementary Figure 4). This approach further ensured stability of the model performance by reducing bias towards certain inner folds where the early stopping function might have halted training prematurely or prolonged it due to specific data characteristics. As a result, we observed increases in validation AUC scores between 0.043 and 0.068 across all four GNN models in DILI prediction in comparison to the hyperparameter optimisation stage (see Table 3). The sequential warm starts method further improved the model performance by progressively training the models on different subsets; inner fold splits, of the training data. This method provided advantages over conventional training approaches by addressing limitations introduced by early stopping functions. When a single validation set is used, models may overfit to specific patterns in the validation set or face temporary plateaus in performance, leading to premature termination which prevent the model from fully capturing the DILI features from the training data. Sequential warm starts addressed this issue by incrementally refining model parameters across the different inner fold splits, ensuring more robust training and improved generalisation. In addition, Wilcoxon Rank Sum Test ensured that the repeated runs in sequential warm starts continued only when significant improvements were observed from the two previous runs. This prevented unnecessary training cycles and prevented overfitting. We observed that there is an increase of 0.132 in test AUC for GraphSAGE model when employing the sequential warm starts method over the conventional methods (see Supplementary Table 3). Overall, our method systematically improved the generalisability and stability of the GNN models during training. Hyperparameter optimisation and random seed reinitialisation laid a solid foundation for model fine-tuning with the sequential warm starts which yielded the best performance metrics across all four GNN models.

### Effectiveness of our custom-designed molecular graphs

Our experimental results demonstrate that the use of custom-designed molecular graphs significantly enhances the performance of GNN models across various prediction tasks. As shown in Table 4, the custom feature sets consistently yielded higher AUC values compared to the basic feature sets across all models and datasets. This consistent improvement underscores the importance of incorporating advanced chemical and structural features in capturing complex molecular characteristics across different chemical spaces, including toxicity (DILI and ClinTox), permeability (BBBP), and biochemical activity (BACE). The enriched feature sets provide the GNN models with more informative and discriminative representations of the molecular graphs by including detailed chemical and structural properties from the 3D optimised molecular representations. Therefore, the models can effectively learn and interpret the underlying patterns associated with the target properties and achieve higher performance, as evidenced by the substantial improvements in AUC scores.

### Comparative analysis of advanced models in molecular property prediction

#### Performance evaluation in DILI prediction

**Table 5:** Comparison of the performance metrics across different molecular property prediction models

Dataset	Model	AUC	ACC	F1	MCC	Input feature space
DILI	DeepDILI [4]	0.659 (N/A)	0.687 (N/A)	0.755 (N/A)	0.331 (N/A)	${\textrm{Chemical}}^{b}$
	SSM [17]	0.691 ± 0.011	0.687 ± 0.005	0.784 ± 0.008	0.338 ± 0.030	${\textrm{Chemical}}^{b}$
	DILIPredictor [18]	0.63 (N/A)	0.590 $\mathrm{(N/A)}^{\dag }$	0.610 (N/A)	0.160 (N/A)	${\textrm{Chemical}}^{b}$ & Biological
	DNN [19]	0.713 (N/A)	0.689 (N/A)	0.753 (N/A)	0.344 (N/A)	${\textrm{Chemical}}^{b}$
	DNN-GATNN [20]	0.757 (N/A)	0.751 (N/A)	0.825 (N/A)	0.399 (N/A)	${\textrm{Chemical}}^{a,b}$
	DILIGeNN (GraphSAGE)*	**0**.**897** ± 0.043	**0**.**836** ± 0.095	**0**.**846** ± 0.106	**0**.**698** ± 0.148	${\textrm{Chemical}}^{a}$
ClinTox	D-MPNN [36]	0.906 ± 0.043	N/A	N/A	N/A	${\textrm{Chemical}}^{a,b}$
	GEM [37]	0.901 ± 0.013	N/A	N/A	N/A	${\textrm{Chemical}}^{a,b}$
	GTOT-Tuning [35]	0.779 ± 0.032	N/A	N/A	N/A	${\textrm{Chemical}}^{a}$
	DGCL [38]	0.971 ± 0.029	N/A	N/A	N/A	${\textrm{Chemical}}^{a,b}$
	HDBind-MoLFormer [41]	**0**.**988** ± 0.000	N/A	N/A	N/A	${\textrm{Chemical}}^{b,c}$
	DILIGeNN (GCN)*	0.918 ± 0.028	**0**.**950** ± 0.009	**0**.**782** ± 0.045	**0**.**603** ± 0.075	${\textrm{Chemical}}^{a}$
BBBP	D-MPNN [36]	0.925 ± 0.036	N/A	N/A	N/A	${\textrm{Chemical}}^{a,b}$
	GEM [37]	0.724 ± 0.004	N/A	N/A	N/A	${\textrm{Chemical}}^{a,b}$
	GTOT-Tuning [35]	0.715 ± 0.008	N/A	N/A	N/A	${\textrm{Chemical}}^{a}$
	DGCL [38]	0.738 ± 0.055	N/A	N/A	N/A	${\textrm{Chemical}}^{a,b}$
	HDBind-MoLFormer [41]	0.992 ± 0.001	N/A	N/A	N/A	${\textrm{Chemical}}^{b,c}$
	DILIGeNN (GraphSAGE)*	**0**.**993** ± 0.006	**0**.**973** ± 0.012	**0**.**982** ± 0.008	**0**.**929** ± 0.029	${\textrm{Chemical}}^{a}$
BACE	D-MPNN [36]	0.898 ± 0.031	N/A	N/A	N/A	${\textrm{Chemical}}^{a,b}$
	GEM [37]	0.856 ± 0.011	N/A	N/A	N/A	${\textrm{Chemical}}^{a,b}$
	GTOT-Tuning [35]	0.853 ± 0.015	N/A	N/A	N/A	${\textrm{Chemical}}^{a}$
	DGCL [38]	0.915 ± 0.168	N/A	N/A	N/A	${\textrm{Chemical}}^{a,b}$
	HDBind-MolCLR [41]	0.824 ± 0.005	N/A	N/A	N/A	${\textrm{Chemical}}^{a,b}$
	DILIGeNN (GraphSAGE)*	**0**.**953** ± 0.020	**0**.**911** ± 0.034	**0**.**906** ± 0.035	**0**.**824** ± 0.068	${\textrm{Chemical}}^{a}$

Models are ordered based on publication date. Metrics in bold represent the highest performance, while those underlined indicate the second highest. All values represented as mean ± std. Different chemical features utilised in each study are superscripted

^*^Our best performing model for DILI, BBBP and BACE prediction tasks is GraphSAGE, and GCN is the best performing model for ClinTox prediction task.

$$^{\dag }$$Balanced Accuracy

^a^Molecular Graph

^b^Chemical descriptors

^c^SMILES embedding

Table 5 summarises the performance of our model compared to recent state-of-the-art models in the DILI prediction task. The models included in the table either use the FDA-released DILI datasets (DILIGeNN, DILIPredictor, SSM, DeepDILI), or combine the FDA-released datasets with LiverTox dataset after processing the additional data to convert DILI classifications into binary labels and remove duplicate compounds (DNN, DNN-GATNN). Other recent state-of-the-art models were excluded from the comparison, as discussed in a later section.

DILIGeNN models benefits from our methodology that includes molecular standardisation and graph generation process. The molecular standardisation ensures data consistency and reduces biases and inaccuracies from tautomeric variability. During the graph generation, we utilised molecular optimisation methods to generate and optimise 3D conformers to a more realistic representation to capture molecular features such as bond lengths, partial charges, and accurate geometric configurations. These features are crucial for accurately predicting DILI outcomes, as they provide detailed information about the molecule’s spatial structure and electron distribution.

Our DILIGeNN model (GraphSAGE) outperforms all other models in the table, achieving the highest AUC of 0.897, surpassing DNN-GATNN’s AUC of 0.757. This demonstrates our model’s ability to effectively distinguish between DILI-positive and DILI-negative compounds, leveraging its graph convolutional architecture to capture complex molecular features.

Unlike the DNN-GATNN ensemble model, which uses 2D molecular graph features together with an additional substructure fingerprint (MACCS) to train DNN-GAT ensemble model [20], DILIGeNN model utilises a single molecular graph representation within a GNN architecture. Despite employing a single GNN architecture without additional chemical fingerprint features, our approach is the most effective by incorporating spatial and electrostatic features in derived from molecular optimisation and model fine-tuning steps.

In addition, the DILIGeNN model not only surpasses other models based solely on chemical data but also outperforms DILIPredictor, which achieves an AUC-PR of 0.79 and AUC-ROC of 0.63 using both chemical and predicted *in vivo* and *in vitro* biological data. Evidence from both DILIPredictor and the other study indicates that incorporating biological features does not consistently improve predictive performance for DILI models [18, 64]. This likely reflects the multifactorial nature of DILI, which involves complex and incompletely understood mechanisms that are difficult to capture through current biological markers [2]. Nevertheless, the approach taken by DILIPredictor represents an important step in integrating chemical and biological data to provide mechanistic insight and improve the interpretability of the model predictions.

By focusing exclusively on chemical features, our graph-based architecture effectively identifies key molecular characteristics associated with DILI prediction. This is particularly advantageous in the early-stage drug development and drug-repurposing phase where DILI-related biological data is often unavailable. While our model focuses on chemical features alone, biological data still play an important role in interpreting the biological processes underlying a model’s prediction. With improved understanding and availability of reliable biological markers, future models could better integrate biological features to enhance predictive performance and improve mechanistic understanding of DILI pathogenesis.

#### Performance evaluation on Clintox, BBBP, and BACE datasets

Table 5 illustrates the performance of our top-performing graph-based models in comparison to the recent state-of-the-art models for the Clintox, BBBP and BACE molecular property prediction tasks. These molecular property prediction tasks are crucial in evaluating a compound’s potential toxicity, permeability and biochemical activity.

DILIGeNN and GTOT-tuning [35] are the only methods that utilise a single molecular graph representation without additional chemical descriptors or molecular embeddings for the prediction tasks and both leverages fine-tuning techniques. All other methods either complement this with chemical descriptors [36–38], or in the case of HDBind-MolFormer [41] combining chemical descriptors with SMILES embedding with language models.

Our model, DILIGeNN (GraphSAGE), achieved AUC scores of 0.993 and 0.953 on the BBBP and BACE datasets, respectively, outperforming the previous state-of-the-art AUC of 0.992 by HDBind-MolFormer [41] for the BBBP and 0.915 by DGCL [38] for the BACE datasets. For the ClinTox dataset, our DILIGeNN (GCN) model with an AUC score of 0.918 ranks third behind HDBind-MolFormer and DGCL.

These results highlight the our model’s capability in capturing molecular features from the molecular graph for permeability and biochemical activity prediction as well as the DILI prediction. This emphasises the importance of integrating advanced chemical feature engineering with the GNN architectures for improved molecular prediction tasks.

### Comparison with other approaches and limitations

Accurate evaluation methodologies are crucial for assessing the performance and generalisability of models, especially in medicinal applications such as DILI prediction. Previous studies by Lim et al., [17] and Li et al., [4] used predefined training and test sets from the DeepDILI dataset, without employing cross-validation to evaluate the entire dataset. Li et al., further divided their training data into two subsets to optimise hyperparameters for their base classifiers, where as Lim et al. used the Therapeutics Data Commons’ DILI benchmark dataset [65] as an external validation dataset [17]. However, this dataset contains drugs originating from the same source as the training dataset (FDA DILIst dataset), and using overlapping data defeats the purpose of the external validation.

In contrast, our study employed the nested CV framework for training and evaluating the GNNs on the DILI dataset, as well as on other molecular property prediction datasets, including Clintox, BBBP, and BACE. Our method uses inner training loops, allowing each instance in the dataset to be used for both training and validation sets. Furthermore, our approach is the only one among current state-of-the-art DILI prediction models that evaluates the entire dataset across outer test splits, providing more reliable assessment of the model’s performance and generalisability.

There are other DILI prediction models that have reported higher performance metrics than those evaluated in this study [66, 67]. However, their reliability may be limited by the use of overlapping DILI datasets and different DILI classification standards. For instance, DILIst is an augmented version of the DILIRank dataset, developed by incorporating other DILI sources [10, 11, 43–45]. These studies integrated data from the overlapping DILI resources without providing sufficient detail on the methods used to identify and remove duplicate compounds or to convert different DILI classifications into a binary label. Such data integration can introduce variability in DILI annotation and increase the risk of data leakage, thereby inflating model performance metrics. In contrast, our study used the FDA’s latest and systematically curated DILIst dataset, providing a consistent and uniform benchmark for model development.

Our approaches have some limitations. In the DILI prediction study, we only make use of chemical features. Biological features are also available and may provide better interpretability of the model as shown with the other approaches [18, 64]. While our method did not achieve the highest performance on the ClinTox dataset, the two methods with better performance used additional chemical features not included in this work. It remains to be investigated whether adding these features could further boost performance for DILIGeNN. While GNNs are explainable by nature, in this work we have not focussed on extracting any information about the decision making process of the models and focussed on classification performance. Recent works on heterogeneous information networks, a type of graph-based method, demonstrate that integrating biological network information, such as drug-protein and protein-disease associations, can improve model interpretability by revealing regulatory pathways and connectivity patterns that underlie drugs’ mechanisms of action [68, 69]. Moreover, generative modelling approaches have not been explored in this study, such as those applied in AMP-Designer to design molecular structures with desirable molecular properties [70]. Similar techniques could be adapted in the future to generate small molecules with reduced DILI risk by training and fine-tuning the model with the DILI dataset.

In ClinTox, BBBP and the BACE prediction study, we could not compare our models with other methods for all metrics as these are not all available, hence we included multiple metrics in our reporting to allow others to compare against DILIGeNN for which we used multiple test set splits in future. In addition, the augmented features are derived from the force field calculations [51] and may not reflect the true molecular representation of the input molecules in their intermolecular interactions with proteins involved in the biological response. However, our study demonstrates that incorporating the augmented features from the 3D optimised structures improves the characterisation of the input molecules compared to traditional molecular graphs from the 2D molecular representation.

We acknowledge that benchmark datasets used in this study may have limitations, including issues with inconsistent chemical representations and invalid molecular structures [71]. To address these concerns, we applied our own molecular standardisation method to improve structural consistency and future reproducibility. The code for our standardisation method, as well as both the original and standardised SMILES strings used in this study, is available in our GitHub.

## Conclusion

In this study, we developed and evaluated four prominent GNN architectures for predicting DILI and other molecular properties using custom-designed graph features as chemical inputs. Our method (DILIGeNN) demonstrated improved performance in comparison to the state-of-the-art methods for DILI, BBBP, BACE prediction tasks. The best performing model in DILI prediction achieved an AUC of 0.897 (GraphSAGE) surpassing DNN-GATNN (AUC = 0.757) that utilises an additional molecular fingerprint with graph-structured data. Additionally, our best performing models in BBBP and BACE (both GraphSAGE) outperformed the state-of-the-art models, advancing molecular property prediction across diverse chemical spaces. These results highlights the effectiveness of DILIGeNN models in capturing complex molecular features with more realistic chemical features, which is crucial for accurate predictions of molecular properties. In addition, our nested CV framework improved predictive performance of GNN models over multiple stages of optimisation and fine-tuning, without losing generalisability.

A key innovation in our approach is the use of the custom-designed graph features, which significantly enhanced the models’ ability to represent and learn from molecular data. By incorporating more realistic molecular features such as bond lengths and partial charges, we have improved performance of DILIGeNN models across different molecular prediction tasks. Our custom feature engineering was crucial for achieving improved performance over current models. Future work involves incorporating an explainability function to extract relevant subgraph structures for each molecular property prediction task.

## Supplementary Information

Supplementary material 1.

## Data Availability

All code and data are provided at https://github.com/tlee23-ic/GNN_DILI.
