# Supplementary material for: Improving drug-induced liver injury prediction using graph neural networks with augmented graph features from molecular optimisation
Source: J Cheminform. 2025 Aug 18;17:124. doi: 10.1186/s13321-025-01068-3 (PMC12359948; doi:10.1186/s13321-025-01068-3)
Supplement: Supplementary file 1 — Supplementary material 1. [file 13321_2025_1068_MOESM1_ESM.pdf]

Supplementary Table 1 Hyperparameter grid search values used during the nested cross-validation for model optimisation

Hyperparameter	Values
Batch Size	{32, 64, 128}
Hidden Channels	{64, 128, 256}
Learning Rate	{0.00001, 0.0001, 0.001}
Dropout Rate	{0.1, 0.2, 0.3, 0.4, 0.5}

The table lists the range of hyperparameters evaluated, including batch size, hidden channel sizes, dropout rates, and learning rates.

Supplementary Table 2 Comparison of the effects of molecular optimisation on bond lengths, with (standardised) and without (original) prior molecular standardisation

Metric (in %)	Original	Standardised
Mean bond length change	2.02 ± 0.62	2.04 ± 0.53
Minimum	0.00	0.45
25th percentile	1.69	1.69
Median	1.96	1.97
75th percentile	2.26	2.30
Maximum	14.44	7.45

The table presents statistical metrics, including mean bond length change, minimum, median, and maximum changes, as well as the 25th and 75th percentiles. Results indicate that molecular standardisation leads to reduced variability in bond length changes and fewer extreme outliers, suggesting a more consistent and reliable optimisation process.

The data used for this analysis, including bond lengths after optimisation of drug molecules, is available in the file `DILIst_bonds.csv` in the supporting documents (GitHub).

The mean bond length change is slightly higher for standardised molecules, which indicates a tighter distribution of bond length changes after molecular standardisation. Importantly, the standard deviation of bond length changes is reduced from 0.62 to 0.53 after standardisation, reflecting decreased variability and increased consistency in molecular geometry.

Moreover, the maximum bond length change is significantly lower for the standardised molecules (7.45%) compared to the non-standardised ones (14.44%), suggesting that extreme variations or outliers are substantially reduced through standardisation. This reduction in extreme values can lead to more reliable predictions and analyses in cheminformatics applications.

A paired t-test was conducted to statistically compare the bond length changes between standardised and non-standardised molecules. The t-statistic was -1.115 with a p-value of 0.265, indicating that the difference in mean bond length changes between the two sets is not statistically significant at the 0.05 significance level. While the mean difference is not significant, the reduction in variability and maximum changes supports the qualitative benefits of molecular standardisation, such as improved data consistency and reliability.

Supplementary Table 3 Comparison of Conventional Training vs Sequential Warm Starts for Graph Neural Network Models.

Model	Method	Validation AUC	Test AUC	Test Accuracy	Test F1	Test MCC
**GCN**	Conventional Training	0.742 ± 0.040	0.702 ± 0.032	0.598 ± 0.018	0.595 ± 0.030	0.274 ± 0.021
	Sequential Warm Starts	0.881 ± 0.062	0.859 ± 0.053	0.750 ± 0.092	0.753 ± 0.112	0.559 ± 0.137
**GAT**	Conventional Training	0.758 ± 0.054	0.712 ± 0.054	0.608 ± 0.051	0.607 ± 0.073	0.283 ± 0.078
	Sequential Warm Starts	0.728 ± 0.054	0.749 ± 0.065	0.647 ± 0.050	0.644 ± 0.069	0.372 ± 0.075
**GIN**	Conventional Training	0.742 ± 0.010	0.706 ± 0.008	0.563 ± 0.034	0.507 ± 0.069	0.271 ± 0.042
	Sequential Warm Starts	0.916 ± 0.051	0.879 ± 0.031	0.806 ± 0.061	0.822 ± 0.064	0.643 ± 0.097
**GraphSAGE**	Conventional Training	0.824 ± 0.061	0.769 ± 0.064	0.645 ± 0.051	0.642 ± 0.061	0.368 ± 0.088
	Sequential Warm Starts	0.953 ± 0.061	0.901 ± 0.038	0.842 ± 0.089	0.854 ± 0.095	0.705 ± 0.143

The table summarises the performance metrics of GCN, GAT, GraphSAGE, and GIN models trained using the Nested CV framework with Sequential Warm Starts compared to conventional training (no splitting into inner folds). Metrics include Validation AUC, Test AUC, Accuracy, F1-score, and MCC, reported as mean ± standard deviation. For Conventional Training, the best seed from model reinitialisation was used, and the training dataset was not further split into inner folds, with all training data used as validation, while employing the same early stopping criteria (patience = 50, delta = 0). Sequential Warm Starts significantly improved all metrics by leveraging progressive learning on training subsets and statistical significance testing to optimise generalisation.

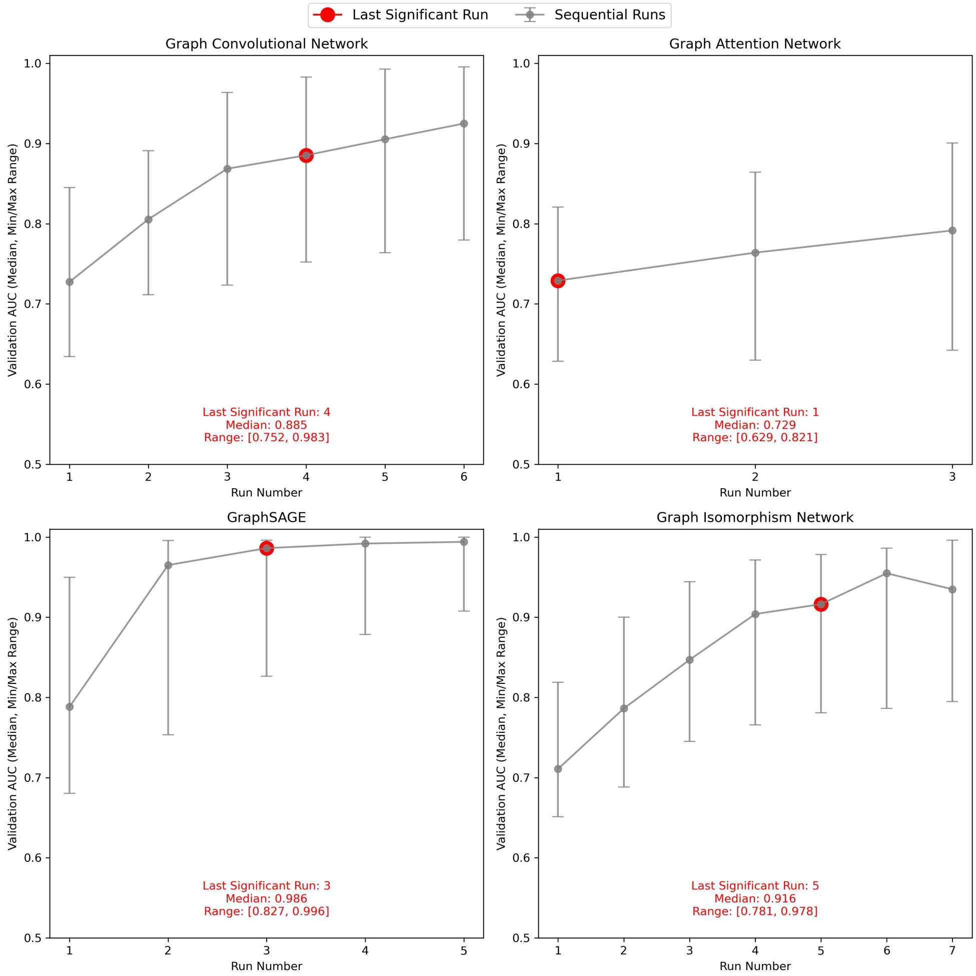

Supplementary Figure 1 Median, Min/Max Range of Validation AUC during sequential warm starts

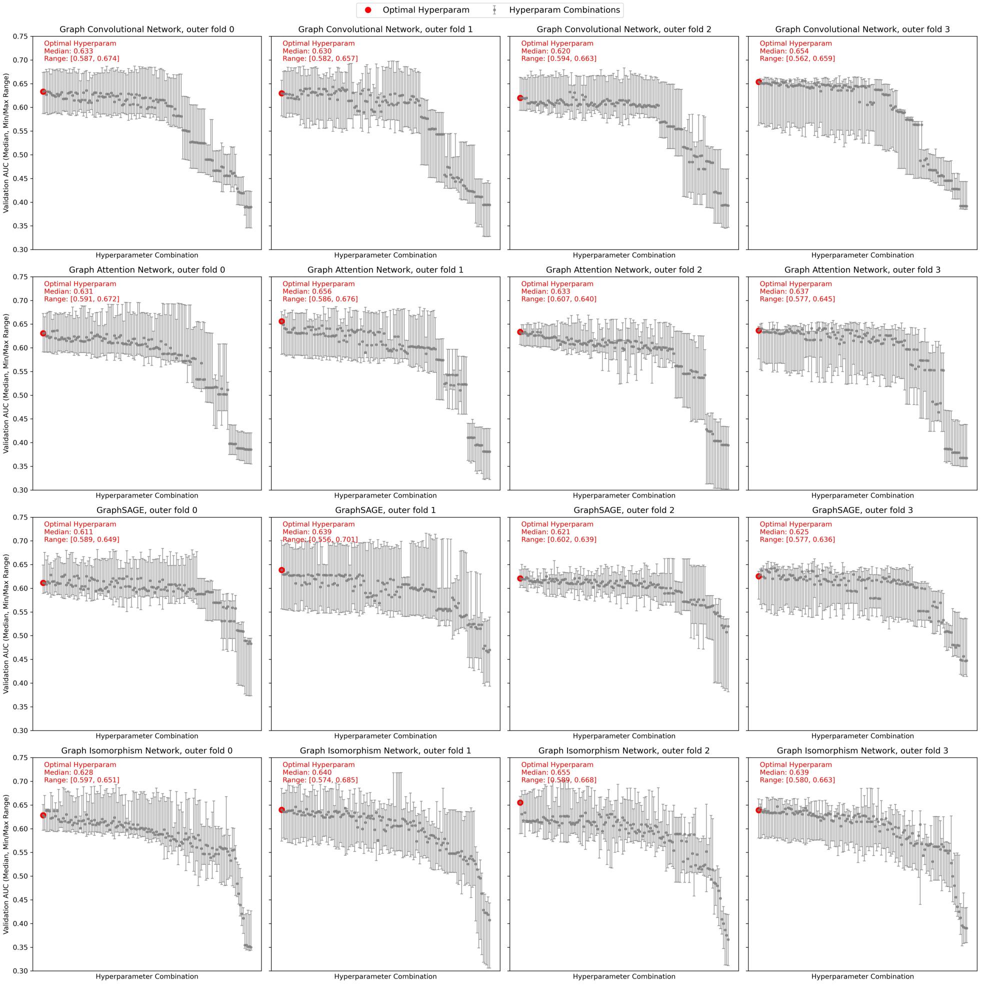

Supplementary Figure 2 Selection of the optimal hyperparameter in the 'Hyperparameter Optimisation' part of the method.

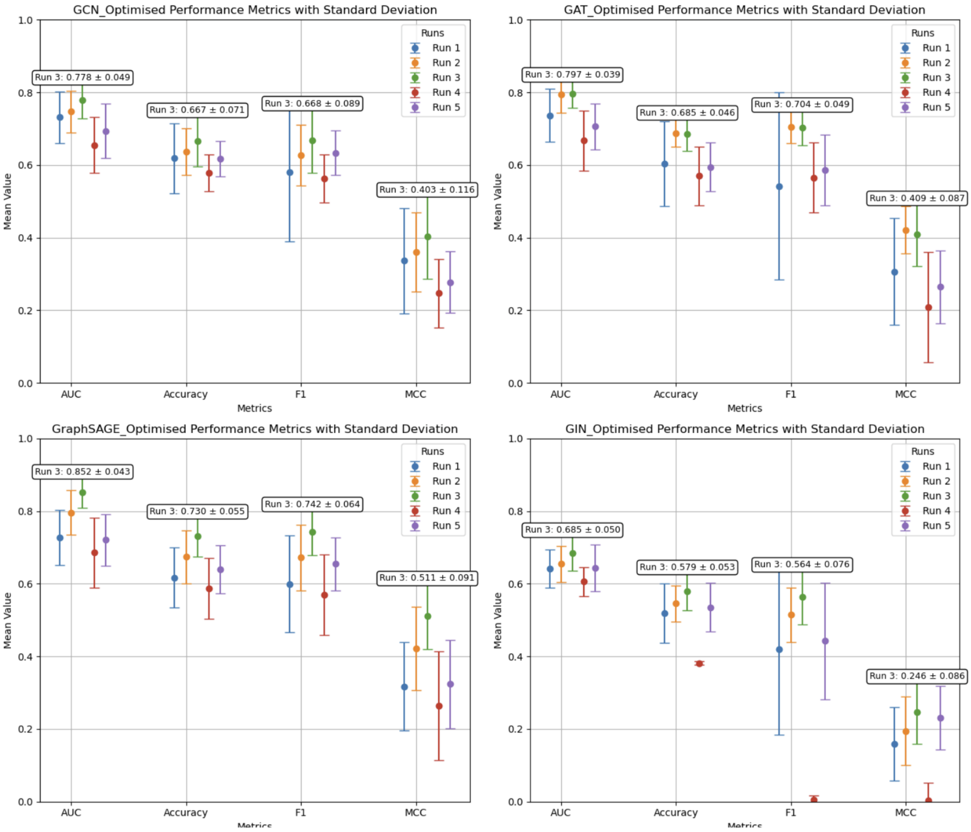

Supplementary Figure 3 Performance analysis of the graph neural network models across multiple runs in the DILI prediction. This figure illustrates the performance analysis of the four graph neural network models: GCN (upper left), GAT (upper right), GraphSAGE (lower left), and GIN (lower right). The metrics used in the analysis are validation AUC, ACC, F1 and MCC. Each dot presents mean and standard deviation of 10 evaluation metric from the 10-fold CV. There are five dots for each metric from five independent runs, with distinct colours and markers to represent each run.

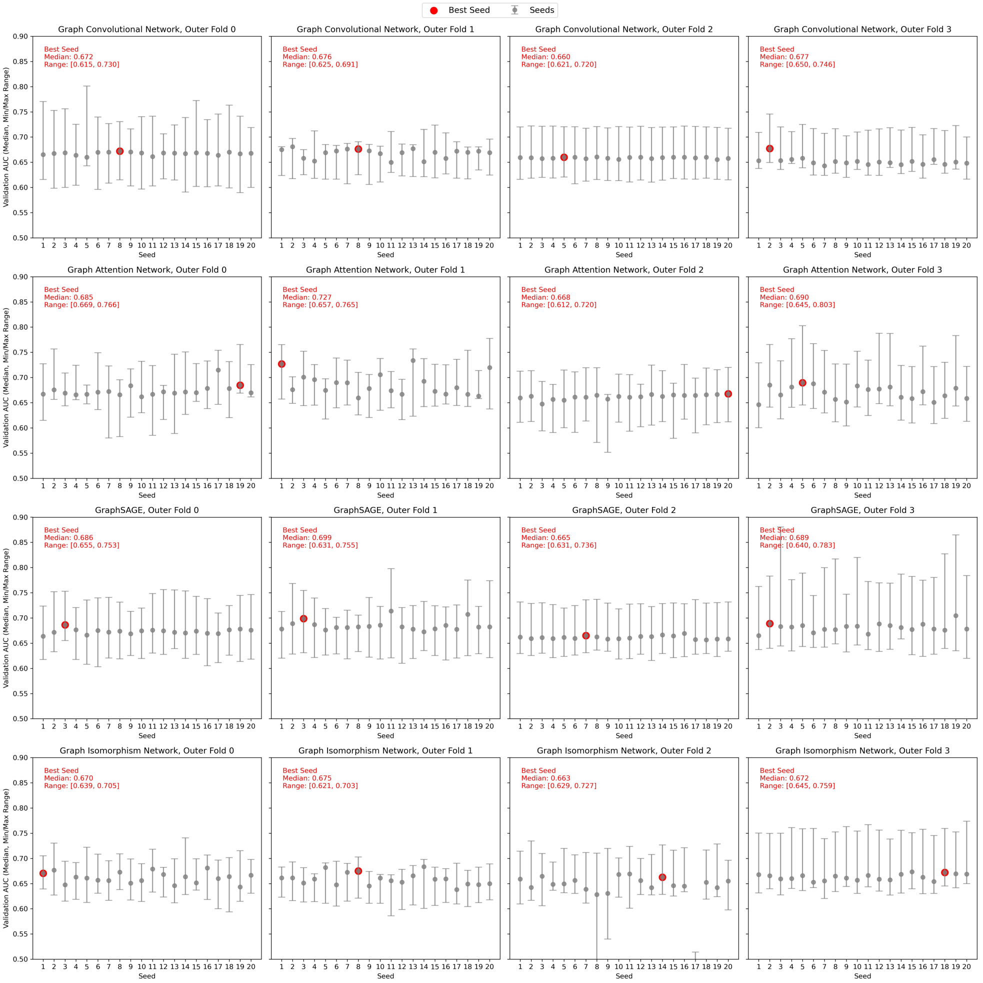

Supplementary Figure 4 Selection of the best seed for model initialisation in the 'Model Reinitialisation' part of the method.
